# AIEgen-Enabled Multicolor Visualization for the Formation of Supramolecular Polymer Networks

**DOI:** 10.3390/molecules27227881

**Published:** 2022-11-15

**Authors:** Shaoyu Xu, Hanwei Zhang, Qingyun Li, Hui Liu, Xiaofan Ji

**Affiliations:** Key Laboratory of Material Chemistry for Energy Conversion and Storage, Ministry of Education, Hubei Key Laboratory of Material Chemistry and Service Failure, Hubei Engineering Research Center for Biomaterials and Medical Protective Materials, School of Chemistry and Chemical Engineering, Huazhong University of Science and Technology, Wuhan 430074, China

**Keywords:** aggregation-induced emission, crosslinking degree, multicolor visualization, supramolecular polymer networks

## Abstract

Extensive reports on the use of supramolecular polymer networks (SPNs) in self-healing materials, controlled release system and degradable products have led more researchers to tap their potential owing to the unique properties. Yet, the attendant efforts in the visualization through conventional luminescence methods during the formation of SPNs have been met with limited success. Herein, we designed a special type of SPNs prepared by **PPMU** polymer chains containing pyrene benzohydrazonate (PBHZ) molecules as AIEgens for the multicolor visualization with naked eyes. The complete detection of the formation process of the networks relied on the PBHZ molecules with aggregation-induced ratiometric emission (AIRE) effect, which enabled the fluorescence of the polymer networks transits from blue to cyan, and then to green with the increasing crosslinking degree derived from the hydrogen bonds between 2-ureido-4-pyrimidone (UPy) units of the polymer chains. Additionally, we certificated the stimuli-responsiveness of the obtained SPNs, and the fluorescence change, as well as observing the morphology transition. The AIEgen-enabled multicolor visualization of the formation of SPNs may provide better understanding of the details of the crosslinking interactions in the microstructural evolution, giving more inspiration for the multifunctional products based on SPNs.

## 1. Introduction

The progressive improvements in function and properties are observed apparently during the crosslinking of primary linear polymer chains into polymer networks [1,2,3]. This is why polymer networks have drawn extensive attention to their applications in biomedical encapsulation and controlled release systems, healable and reprocessable materials, etc. [2,4,5,6,7,8]. In terms of the interconnecting bonds, polymer networks can be classified into covalent networks and supramolecular polymer networks (SPNs) [9,10,11,12]. Covalent networks are produced by polymer chains through permanent covalent bonds, while SPNs are based on non-covalent interactions [13,14]. When covalent networks are utilized to construct tough materials for their strong crosslinks, SPNs are favored due to their unique self-assembly capability, reversibility and stimuli-responsiveness provided by the weak crosslinking motifs such as hydrogen bonding, metal–ligand coordination, host–guest interactions and π–π interactions [15,16,17,18,19,20,21,22,23]. Sanjayan and co-workers proposed supramolecular polymer networks cross-linked by Janus-faced hydrogen bonds, which combine the advantages of recyclability, stability and reprocessability [24]. Zhao and co-workers prepared Eu^3+^- and Tb^3+^-containing hydrogels through metal−ligand coordination for smart confidential information protection. Huang and co-workers realized time-dependent information encryption through the construction of self-assembled supramolecular host–guest networks comprised of pillar [5] arene host and nitrile guest [25]. Therefore, it is of great necessity to monitor the formation of the supramolecular polymer networks aimed at creating more customized functional products [26].

Fluorescence techniques as prevailing labeling methods are considered rationally; their unique properties, superior to conventional nonfluorescent labeling, guarantee their bright future in the visualization of microscopic processes such as the monitoring of the formation of the cross-linked polymeric network [27,28,29,30,31,32]. Specifically, there are two types of fluorophores applied in fluorescence techniques [33,34,35,36,37,38,39,40]. One type of fluorophores includes fluorophores with an aggregation-caused quenching (ACQ) effect in which luminescence is quenched in the aggregate state [35,39,40]. The other fluorophores with an aggregation-induced emission (AIE) effect become emissive as a result of the aggregation process [33,34,36,37,38]. As above, ACQ/AIE-active fluorophores are introduced into SPNs, with the increasing crosslinking degree, the aggregation state caused by the polymer chains close to each other has different effects on the luminescence of networks [41,42]. The luminescence of AIE-active fluorophores-based SPNs can be fulfilled at high crosslinking levels, but it fails at low levels, while ACQ networks behave conversely [41]. In spite of the opposite luminous mechanism, both of the networks consisting of the two mentioned fluorophores, when applied in the detection of the crosslinking, just exhibit fluorescence “ON” at certain limited crosslinking degree with constant emission wavelength, which sets the limits for the integrity of the monitoring process of the crosslinking as well as the indicative meaning of the fluorescence color changes.

**Scheme 1:**
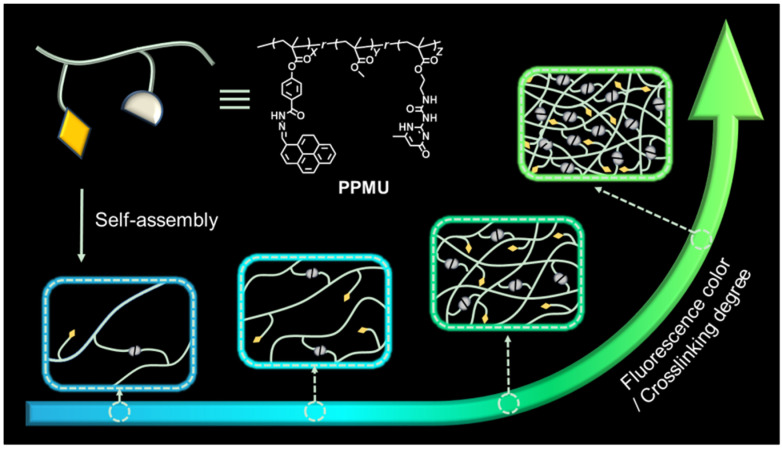
Chemical structure of polymer chain **PPMU**, and cartoon representations of its proposed crosslinking process into supramolecular polymer networks and the change of fluorescence color with increasing concentrations of **PPMU** solutions.

Given this situation, exploiting the potentially satisfying molecules capable of circumventing the problem to realize perfect visualization is urgently needed. In recent years, the structure tunable pyrene benzohydrazonate-based (PBHZ-based) molecular platform has been reported as one of the research hotspots benefiting from its distinctive feature of aggregation-induced ratiometric emission (AIRE)—an uncommon AIE phenomenon that emission wavelength changes with the increasing aggregation degree of the molecule [43,44,45,46]. The PBHZ fluorophore was formed by linking pyrene with isolated benzene. It owned the photophysical properties of pyrene at low concentrations, and as the concentration increased, the formation of intermolecular hydrogen-bonding in the aggregation state led to fixation of the molecular conformations, giving rise to longer-wavelength emission of the fluorophore [47,48]. We envision that the introduction of the special molecule instead of other conventional AIEgens into the polymerization system may contribute to the improvement of the detection process. In this work, we put forward a strategy of advanced multicolor visualization to reveal the formation of supramolecular polymer networks (SPNs) relying on AIE fluorophores (Scheme 1). SPNs originated from the self-assembly of the polymers **PPMU**, which consisted of AIEgen pyrene benzohydrazonate (PBHZ), poly(methyl methacrylate (PMMA) main chains and functionalized 2-ureido-4-pyrimidone (UPy) units. In the evolution of polymers to SPNs, the reduction of the distance between polymers provided more opportunities for the UPy units to interact based on the multiple hydrogen bonding, leading to the increasing aggregation degree of the fluorescent molecules as well as crosslinking degree until SPNs are formed. At the same time, PBHZ fluorophores of the polymers got close to each other, their changed intermolecular aromatic stacking distances in the aggregation state endowed SPNs with the variation of fluorescence colors from blue to green. On the basis of the luminescence principle, the PBHZ-based SPNs not only emitted fluorescence at broad cross-linking degree, but also owned discriminative power to indicate the cross-linking degree through the change of the fluorescence colors.

## 2. Results and Discussion

### 2.1. Evidence of SPNs Formation

**Figure 1:**
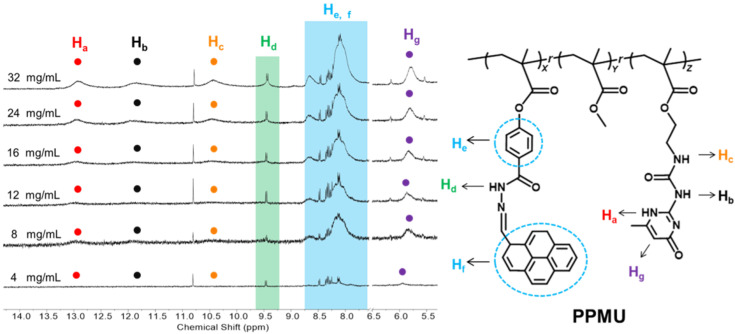
Partial ^1^H NMR spectra (400 MHz, CDCl_3_, 298 K) of **PPMU** at different concentrations (4 mg/mL, 8 mg/mL, 12 mg/mL, 16 mg/mL, 24 mg/mL, 32 mg/mL).

As shown in Figure 1, there were stacked spectra for six ^1^H NMR spectroscopies of **PPMU** in CDCl_3_ (solution) in ascending order of concentrations from 4 mg/mL to 32 mg/mL [49,50]. The signals of H_a_, H_b_, H_c_ and H_g_ belonged to the UPy units of the **PPMU** polymer chains, while the peaks marked with H_d_, H_e_,_f_ corresponded to the PBHZ molecules. Through a vertical comparison, the signal enhancement of H_a_ was observed obviously with the increasing concentrations. The same occurred in the signals of H_b_ and H_c_, additionally, both of which shifted to high-field. The proton signal H_b_ shifted from 12.02 ppm to 11.83 ppm and H_c_ shifted from 10.51 ppm to 10.44 ppm. The changes of three signals disclosed the hydrogen bonding interactions between the UPy units in the high concentrations. The signals H_d_ and H_e,f_ circled by the squares had increased peak widths at high concentrations, which were caused by the hydrogen bonding originating from the close stacking of PBHZ molecules. Accordingly, the aggregation status of the mentioned units as well as the formation of SPNs can be reflected by the ^1^H NMR spectroscopies.

**Figure 2:**
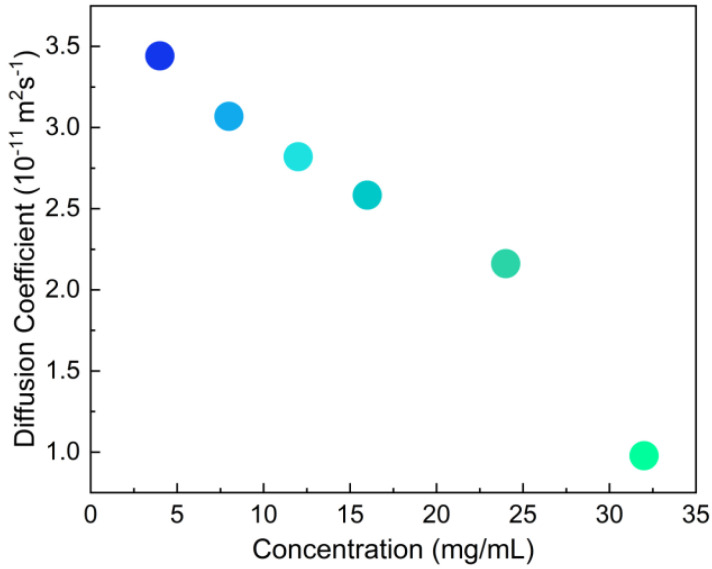
Diffusion coefficients of **PPMU** at 298 K in CDCl_3_ at different concentrations.

DOSY experiments were used to explore the flowability of the polymers in CDCl_3_ at different concentrations [51]. The diffusion coefficients of **PPMU** solutions over concentration range from 4 mg/mL to 32 mg/mL were recorded in Figure 2. It was clear that as the concentrations increased from 4 mg/mL to 32 mg/mL, the diffusion coefficients of the polymer solutions decreased gradually from 3 × 10^−11^ m^2^ s^−1^ to less than 1 × 10^−11^ m^2^ s^−1^. On the basis of the phenomenon that mobility was inversely proportional to concentration, these closer **PPMU** polymer chains in the high concentrations might prompt more non-covalent crosslinking through hydrogen bonds between the UPy units. SPNs exhibited lower mobility compared with the linear polymer chains capable of free movement.

**Figure 3:**
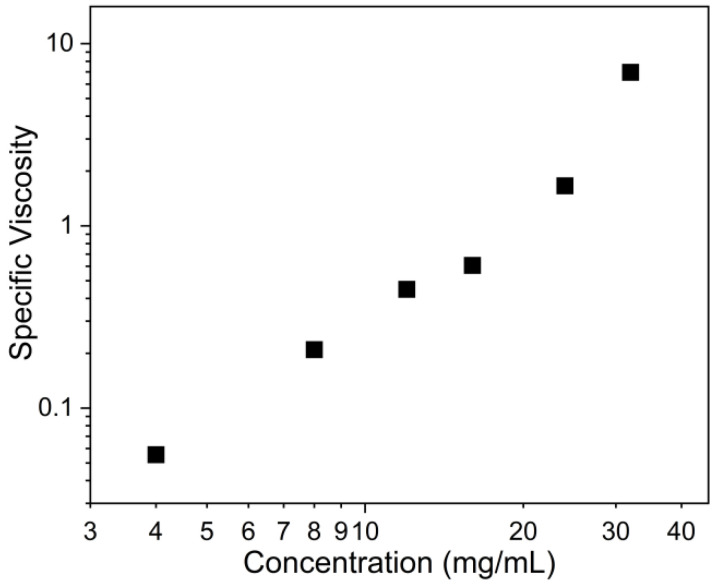
Special viscosities of **PPMU** at 298 K in CHCl_3_ at different concentrations.

Then, we tried to discover how the viscosities of **PPMU** solutions change with increasing concentrations as a way of demonstration of SPNs formation [49]. An Ubbelohde viscometer was used to determine the data and to clarify the flow resistance of the polymer solutions at various concentrations. As shown in Figure 3, the special viscosities of the **PPMU** solutions at a concentration of 32 mg/mL was approximately ten times higher than the solutions at 4 mg/mL, and the viscosities rose with the increasing concentrations in the determination process. The rise in flow resistance of the **PPMU** solutions reflected the microstructure evolved from polymer chains to SPNs since the increasing crosslinking degree of the polymer solutions caused the difficulties in the flow process.

The above characterizations, by ^1^H NMR, viscosity and DOSY experiments, strongly supported more crosslinking interactions between polymer chains to form SPNs as the concentrations increase.

### 2.2. Visualization of SPNs Formation Process

**Figure 4:**
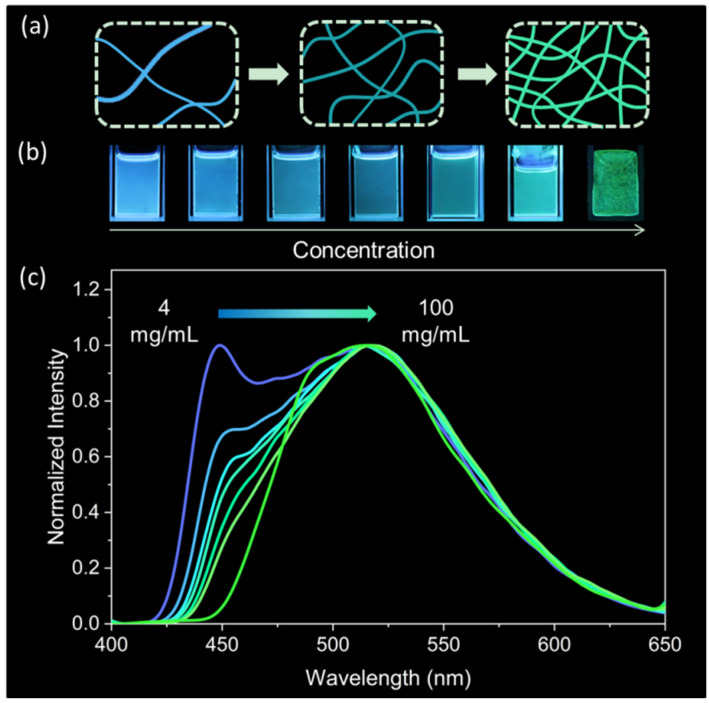
(**a**) Schematic illustration of cross-linked polymers during the formation of the supramolecular polymer networks with the increasing concentrations of **PPMU** solutions; (**b**) Fluorescent photographs and (**c**) Normalized fluorescent spectra of **PPMU** at different concentrations (4 mg/mL, 8 mg/mL, 12 mg/mL, 16 mg/mL, 24 mg/mL, 32 mg/mL, 100 mg/mL) in CHCl_3_ at 298 K. λ_ex_ = 365 nm. Slit: 10/8 nm.

It is envisioned that the key point of the multicolor visualization lies in the special AIEgen fluorophore PBHZ molecules with AIRE effect. As the concentration increased from 4 mg/mL to 100 mg/mL, spatial constraints brought polymer chains close together, naturally resulting in the aggregation state of the UPy units and PBHZ fluorophore. When the UPy units were responsible for non-covalent crosslinking through hydrogen bonds, the PBHZ molecules worked on the fluorescence color changes of the polymer solutions with the help of the AIRE effect (Figure 4a).

Thus, the fluorescence colors of the polymer solutions were monitored at different concentrations from 4 mg/mL to 100 mg/mL under 365 nm UV light in Figure 4b. The blue color can be observed with the naked eye at low concentrations of 4–12 mg/mL, subsequently experiencing a transition to the cyan color at medium concentrations of 16–32 mg/mL, and then changing to green color at high concentration of 64 mg/mL. To reconcile with the observations, the normalized fluorescent spectra (Figure 4c) had been recorded over the same concentration range. Treated with the same excitation wavelength of 365 nm, significant redshifts from 450 nm to 515 nm were observed in the emission maximum of the polymers when concentrations increased.

**Figure 5:**
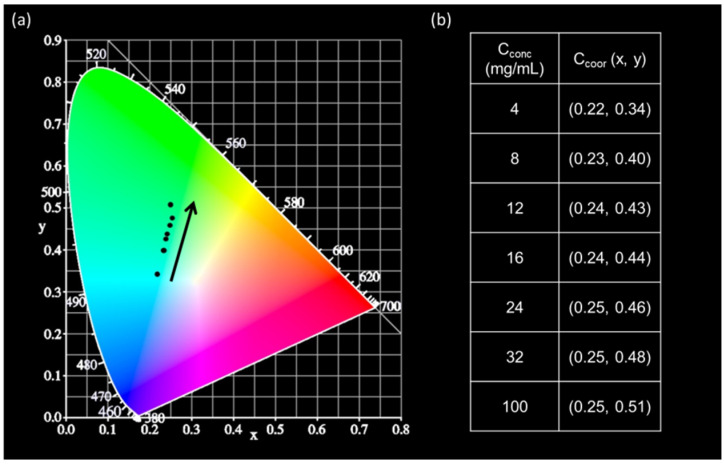
(**a**) The corresponding CIE coordinates of **PPMU** at different concentrations (4 mg/mL, 8 mg/mL, 12 mg/mL, 16 mg/mL, 24 mg/mL, 32 mg/mL, 100 mg/mL) in CHCl_3_; (**b**) Table of the corresponding relationship between **PPMU** concentrations (C_conc_) and CIE coordinates (C_coor_).

In addition, the consistent approach of CIE chromaticity coordinate diagram (Figure 5a) was adopted, providing the corresponding CIE coordinates of the solutions at different concentrations. Looking in the direction indicated by the black arrow, the locations of CIE coordinates moved to the upper right corner gradually with the increasing concentrations, whose corresponding colors coincided with what human eye perceives. The detailed CIE coordinates affected by the concentrations are listed in Figure 5b, and on it, it can be found that the value x increased from 0.22 to 0.25, while the value y increased from 0.34 to 0.51.

Apart from the photographs, the measurements of the fluorescent spectra and CIE chromaticity diagram with CIE coordinates also demonstrated the feasibility of the visualization during the formation of SPNs at high crosslinking degree. As was stated above, the synchronicity between the crosslinking degree and fluorescent colors fulfilled the multicolor visualization equipped with the distinguished power to judge the crosslinking degree by the color of the polymers.

### 2.3. Stimuli-Responsiveness of SPNs

**Figure 6:**
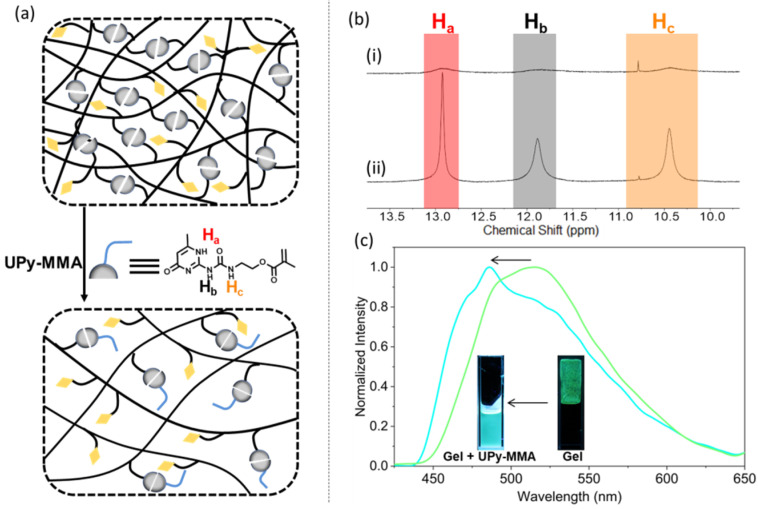
(**a**) Schematic diagram of the depolymerization process of supramolecular polymers networks when **UPy-MMA** was added; (**b**) The comparison of partial ^1^H NMR spectra (400 MHz, CDCl_3_, 298 K) of (i) **PPMU** (100 mg/mL) (ii) **PPMU** (100 mg/mL) added with **UPy-MMA** (**PPMU** and **UPy-MMA**’s final concentrations were 100 mg/mL and 35 mg/mL, respectively); (**c**) Normalized fluorescent spectra of **PPMU** (100 mg/mL) and **PPMU** added with **UPy-MMA** (**PPMU** and **UPy-MMA**’s final concentrations were 100 mg/mL and 35 mg/mL, respectively) at 298 K in CHCl_3_. λ_ex_= 365 nm. Slit: 10/8 nm. Inset: Fluorescent photographs of **PPMU** in CHCl_3_ before and after the addition of **UPy-MMA** recorded under a handheld UV lamp.

Stimuli-responsiveness is regarded as one of symbolic properties of SPNs. To demonstrate the significant characteristic, **UPy-MMA** monomers were added to the prepared SPNs. We anticipated that the wandering **UPy-MMA** molecules participated in the interactions between the UPy units of the polymer chains as strong competitors. The UPy units of **PPMU** chains in the original dimerization turned to form hydrogen bonds with the free **UPy-MMA** molecules, leading to the disassociation of SPNs with the reduced hydrogen bonding sites between polymer chains (Figure 6a) [52]. The ^1^H NMR spectra of SPNs before (Figure 6bi) and after (Figure 6bii) the addition of the **UPy-MMA** monomers were given to support the hypothesis. It could be found that the intensity of the proton signal of H_a_, H_b_, H_c_, owned by the UPy structures, were lower than the solutions fixed with free **UPy-MMA** molecules. Furthermore, all of the three signals varied from broad peaks to narrow peaks after the addition. Both of the changes suggested the hydrogen bonds for the construction of SPNs had been destroyed by the excessive **UPy-MMA** monomers. As shown in Figure 6c, the green gel referring to SPNs of high crosslinking degree, suffering from the damage of the hydrogen bonds between the polymer chains, transformed to the blue liquid on behalf of SPNs of low crosslinking degree. The fluorescent spectra of the solutions before and after treated with the stimulus are shown in Figure 6c as well, being surprisingly in agreement with the color change. There was a blue shift between the maximum emission wavelengths of SPNs from 515 nm to 485 nm.

## 3. Materials and Methods

### 3.1. Reagents and Chemicals

The reagents and chemicals used were commercially available from suppliers.

### 3.2. Synthesis of PPMU Polymer

The component units making up **PPMU** polymers included methyl methacrylate, PBHZ molecules (compound **3**) and UPy units (compound **4**), whose respective diagrams of synthetic routes were given in the Supplementary Materials (Schemes S1–S3, Figures S1–S14, Table S1).

#### 3.2.1. Synthesis of PBHZ Molecule

The preparation of PBHZ molecule (compound **3**) proceeded in three steps along with intermediate products compound **1**, compound **2**.

Compound **1**: Methyl 4-hydroxy benzoate (9.12 g, 60.0 mmol) was added to Hydrazine hydrate (46.5 mL, 960 mmol) and the mixture refluxed overnight. The solid obtained by filtration was washed with hexane to obtain compound **1**.

Compound **2**: Compound 1 (3.80 g, 25.0 mmol) and pyrene-1-carbaldehyde (3.80 g, 25.0 mmol) were dissolved in methanol (200 mL) in a 500 mL round-bottom flask at room temperature. Glacial acetic acid (0.625 mL) was added to the well-stirred solution. The temperature of the mixture was raised to 80 °C. After 5 h, the obtained solid was washed with methanol to obtain compound **2**.

Compound **3**: In a 500 mL round-bottom flask, compound **2** (625 mg, 1.71 mmol) and TEA (347.5 mg, 3.44 mmol) were mixed well in CH_2_Cl_2_ (200 mL) at room temperature. After the dropwise addition of Methacryloyl chloride (207.5 mg, 2 mmol) at 0 °C, this reaction lasted for 12 h at room temperature. Then, CH_2_Cl_2_ was removed from the mixture through vacuum filtration to acquire the solid, which was purified by column chromatography (silica gel, CH_2_Cl_2_: methanol = 300:1, *v*/*v*, eluent) to obtain compound **3** [53].

#### 3.2.2. Synthesis of UPy Unit

The solution of 6-Methylisocytosine (7.34 g, 58.6 mmol) in DMSO was heated to 170 °C with an oil bath. Then, 2-isocyanatoethyl methacrylate (ICEMA) (10.0 g, 64.5 mmol) was added immediately to the solution with water bath instead of oil bath just in case of a vigorous reaction where the molecules were quenched quickly for the polymerization. The pure compound **4** was obtained after the precipitated solid was washed with cyclohexane and dried under reduced pressure [54].

#### 3.2.3. Synthesis of PPMU Polymer Chain

The **PPMU** polymer chain was obtained by the free radical polymerization of compound **3**, compound **4** and methyl methacrylate. Compound **4** (841 mg, 3.00 mmol), compound **3** (32.5 mg, 0.075 mmol) and methyl methacrylate (3.75 g, 37.5 mmol) were dissolved in 30 mL DMSO, followed by the addition of azobisisisobutyronitrile (AIBN) (9.25 mg, 0.056 mmol), immediately followed by a stream of nitrogen (N_2_) bubbling through the reaction mixture for 15 min. Then, the solution was heated to 80 °C and stirred continuously for 10 h. The reaction was stopped by freezing the reaction mixture in ice water. The resulting solution was added to methanol (3 × 300 mL) and then filtered through vacuum to obtain **PPMU** polymers.

### 3.3. Preparation of PPMU Solutions in CHCl_3_

Afterwards, a series of **PPMU** solutions in CHCl_3_ were prepared at different concentrations (4 mg/mL, 8 mg/mL, 12 mg/mL, 16 mg/mL, 24 mg/mL, 32 mg/mL, 100 mg/mL) by mixing different masses of **PPMU** polymers with chloroform.

### 3.4. Characterization

In the characterization stage, ^13^C NMR spectra and ^1^H NMR spectra were recorded with a Bruker Advance 400 MHz spectrometer at 298 K. High-resolution electrospray ionization mass spectra (ESI-MS) were recorded with a Bruker microOTOF II. Gel permeation chromatography (GPC) measurements were carried out on an Elite P230pII Elite HPLC system in tetrahydrofuran (THF). Fluorescent emission spectra were measured with a Perkin Elmer LS55 fluorescence spectrophotometer at 298 K. The DOSY experiments were based on the ^1^HNMR spectroscopy. The viscosity data were obtained through Ubbelohde viscometer using chloroform as solvent at room temperature.

Compound **1** yield: 6.84 g; 75%; white solid. ^1^H NMR (DMSO-*d*_6_, 400 MHz, 298 K) δ 9.74 (brs, 1H), 9.49 (s, 1H), 7.68 (d, J = 8.5 Hz, 2H), 6.77 (d, J = 8.3 Hz, 2H), 4.37 (s, 2H). ^13^C NMR (DMSO-*d*_6_, 100 MHz, 298 K) δ 166.36, 160.44, 129.26, 124.42, 115.27. HRMS (ESI^+^) Calcd for C_7_H_8_N_2_O_2_ [M+H]^+^: 153.0659, found: 153.0870.

Compound **2** yield: 6.22 g; 68%; orange solid. ^1^H NMR (DMSO-*d*_6_, 400 MHz, 298 K) δ 11.90 (s, 1H), 10.23 (s, 1H), 9.53 (s, 1H), 8.83 (d, J = 9.4 Hz, 1H), 8.60 (d, J = 8.2 Hz, 1H), 8.37 (d, J = 7.9 Hz, 4H), 8.25 (q, J = 8.9 Hz, 2H), 8.13 (t, J = 7.6 Hz, 1H), 7.94 (d, J = 8.7 Hz, 2H), 6.96 (d, J = 8.7 Hz, 2H). ^13^C NMR (DMSO-*d*_6_, 100 MHz, 298 K) δ 163.18, 161.28, 146.02, 132.24, 131.34, 130.63, 130.19, 129.15, 128.77, 127.90, 127.67, 127.07, 126.53, 126.19, 125.73, 125.37, 124.64, 124.28, 122.91, 115.61. HRMS (ESI^+^) Calcd for C_24_H_16_N_2_O_2_ [M+H]^+^: 365.1285, found: 365.1194.

Compound **3** yield: 207.2 mg; 28%; yellow solid. ^1^H NMR (DMSO-*d*_6_, 400 MHz, 298 K) δ 12.12 (s, 1H), 9.53 (s, 1H), 8.83 (d, J = 9.4 Hz, 1H), 8.60 (d, J = 8.2 Hz, 1H), 8.38 (d, J = 7.6 Hz, 4H), 8.26 (q, J = 8.9 Hz, 2H), 8.11 (dd, J = 19.2, 8.1 Hz, 3H), 7.43 (d, J = 8.6 Hz, 2H), 6.34 (s, 1H), 5.96 (s, 1H), 2.04 (s, 3H). ^13^C NMR (DMSO-*d*_6_, 100 MHz, 298 K) δ 165.53, 162.74, 153.71, 147.13, 135.57, 132.45, 131.50, 131.34, 130.63, 129.69, 129.29, 129.15, 128.93, 128.69, 127.90, 127.39, 127.12, 126.63, 126.28, 125.75, 125.56, 124.63, 124.25, 122.89, 122.57, 18.50. HRMS (ESI^+^) Calcd for C_28_H_20_N_2_O_3_ [M+H]^+^: 433.1547, found: 433.1143.

Compound **4** yield: 15.75 g; 96%; white solid. ^1^H NMR (CDCl_3_, 400 MHz, 298 K) δ 12.96 (s, 1H), 11.95 (s, 1H), 10.50 (s, 1H), 6.17 (s, 1H), 5.78 (s, 1H), 5.54 (s, 1H), 4.26 (t, J = 5.7 Hz, 2H), 3.57 (q, J = 5.7 Hz, 2H), 2.23 (s, 3H), 1.93 (s, 3H). ^13^C NMR (CDCl_3_, 100 MHz, 298 K): 172.85, 167.33, 156.75, 154.48, 148.32, 136.11, 125.83, 106.69, 63.07, 38.76, 18.95, 18.29. HRMS (ESI^+^) Calcd for C_12_H_16_N_4_O_4_ [M+Na]^+^: 303.1069, found: 303.1123.

**PPMU** polymer: ^1^H NMR (400 MHz, CDCl_3_) δ 12.89 (s, NH), 11.79 (s, NH), 10.40 (s, NH), 9.39 (d, NH), 8.60–8.02 (m, CH), 5.74 (s, CH), 4.33–3.71 (m, CH_2_), 3.53 (s, OCH_3_). The ratio of x/y/z was (1)/(66.87/3)/(4.06), namely, 1/22.29/4.06.

## 4. Conclusions

In summary, we realized the intuitive multicolor visualization to monitor the formation of SPNs by the introduction of fluorophores with AIRE effect into the **PPMU** polymer chains, which were composed of AIEgens pyrene benzohydrazonate (PBHZ), poly(methyl methacrylate (PMMA) main chains and functionalized 2-ureido-4-pyrimidone (UPy) units. A series of **PPMU** solutions at different concentrations represented the incremental crosslinking degree in the evolution of polymer system from polymer chains to SPNs, where the UPy units of the polymer chains played a crucial role through the multiple-hydrogen-bonding arrays. Meanwhile, the increasing aggregation degree of PBHZ AIEgens accompanied with the polymer chains becoming closer to each other, due to the AIRE effect, allowing the fluorescence color change of SPNs from blue to green with the increasing crosslinking degree. Furthermore, we verified the stimuli-responsiveness of the prepared SPNs by the addition of the free UPy-MMA molecules.

The method facilitates the visualization of the formation process of SPNs regardless of whether the crosslinking degree is high or not, and performs the optimization in the recognition of the crosslinking degree through the fluorescence colors. We believe that the strategy opens up new vistas for the rational design of SPNs through a deeper understanding of the formation process of the networks, leading to improved functional materials.

## Data Availability

All data relevant to the publication are included.

## Supplementary Materials

The following supporting information can be downloaded at: https://www.mdpi.com/article/10.3390/molecules27227881/s1, Scheme S1: Synthetic route of compound **3**; Scheme S2: Synthetic route of compound **4**; Scheme S3: Synthetic route of **PPMU** polymer; Figure S1: ^1^H NMR spectrum (DMSO-*d*_6_, 400 MHz, 298 K) of **1**; Figure S2: ^13^C NMR spectrum (DMSO-*d*_6_, 100 MHz, 298 K) of **1**; Figure S3: HR-ESI^+^-MS spectrum of **1**; Figure S4: ^1^H NMR spectrum (DMSO-*d*_6_, 400 MHz, 298 K) of **2**; Figure S5: ^13^C NMR spectrum (DMSO-*d*_6_, 100 MHz, 298 K) of **2**; Figure S6: HR-ESI^+^-MS spectrum of **2**; Figure S7: ^1^H NMR spectrum (DMSO-*d*_6_, 400 MHz, 298 K) of **3**; Figure S8: ^13^C NMR spectrum (DMSO-*d*_6_, 100 MHz, 298 K) of **3**; Figure S9: HR-ESI^+^-MS spectrum of **3**; Figure S10: ^1^H NMR spectrum (CDCl_3_, 400 MHz, 298 K) of **4**; Figure S11: ^13^C NMR spectrum (CDCl_3_, 100 MHz, 298 K) of **4**; Figure S12: HR-ESI^+^-MS spectrum of **4**; Figure S13: ^1^H NMR spectrum (CDCl_3_, 400 MHz, 298 K) of **PMMU** polymer; Figure S14: GPC trace of **PPMU** polymer; Table S1: GPC analysis of **PPMU** polymer using conventional calculations, with poly-styrene as the standard and THF as the solvent.

## Sample Availability

Not available.
