# Supplementary material for: AIEgen-Enabled Multicolor Visualization for the Formation of Supramolecular Polymer Networks
Source: Molecules. 2022 Nov 15;27(22):7881. doi: 10.3390/molecules27227881 (PMC9695632; doi:10.3390/molecules27227881)
Supplement: Supplementary file 1 [file molecules-27-07881-s001.zip › molecules-2031658-supplementary.pdf]

Supplementary Materials

AIEgen-Enabled Multicolor Visualization for the Formation of Supramolecular Polymer Networks

Shaoyu Xu, Hanwei Zhang, Qingyun Li, Hui Liu and Xiaofan Ji *

Key Laboratory of Material Chemistry for Energy Conversion and Storage, Ministry of Education, Hubei Key Laboratory of Material Chemistry and Service Failure, Hubei Engineering Research Center for Biomaterials and Medical Protective Materials, School of Chemistry and Chemical Engineering, Huazhong University of Science and Technology, Wuhan 430074, China

* Correspondence: xiaofanji@hust.edu.cn

Citation: Xu, S.; Zhang, H.; Li, Q.; Liu, H.; Ji, X. AIEgen-Enabled Multicolor Visualization for the Formation of Supramolecular Polymer Networks. *Molecules* **2022**, *27*, 7881. https://doi.org/10.3390/molecules27227881

Academic Editor: Youhong Tang

Received: 30 October 2022

Accepted: 11 November 2022

Published: 15 November 2022

Publisher's Note: MDPI stays neutral with regard to jurisdictional claims in published maps and institutional affiliations.

Copyright: © 2022 by the authors. Submitted for possible open access publication under the terms and conditions of the Creative Commons Attribution (CC BY) license (https://creativecommons.org/licenses/by/4.0/).

1. Synthesis and characterization of compound 3.

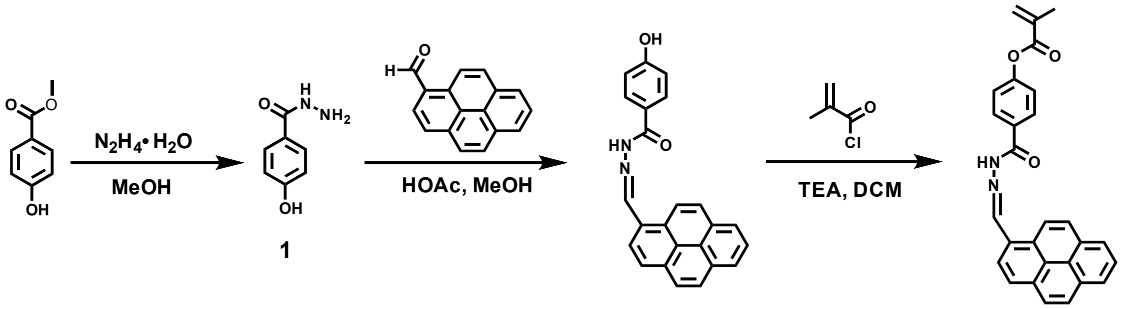

Scheme S1. Synthetic route of compound **3**.

Characterization of compound 1.

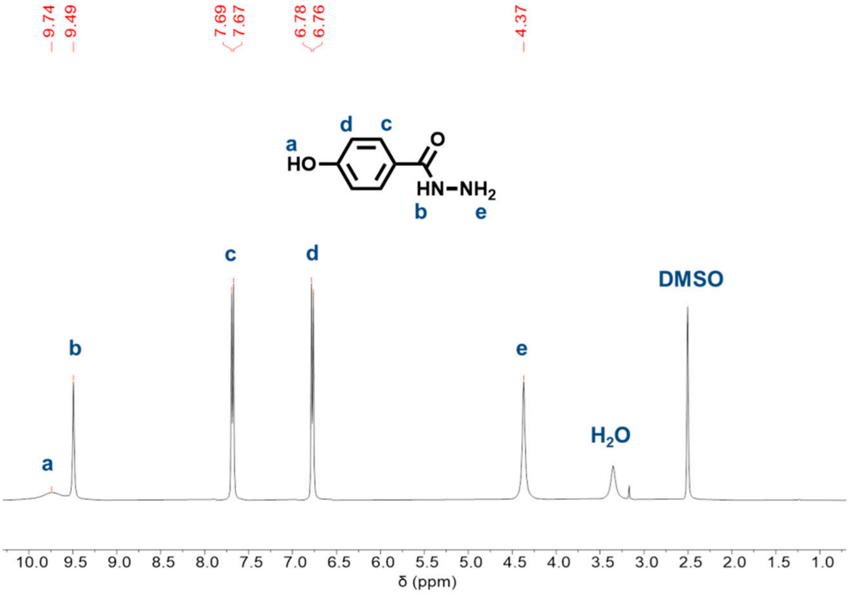

Figure S1. ^{1}H NMR spectrum (DMSO-*d*₆, 400 MHz, 298 K) of **1**.

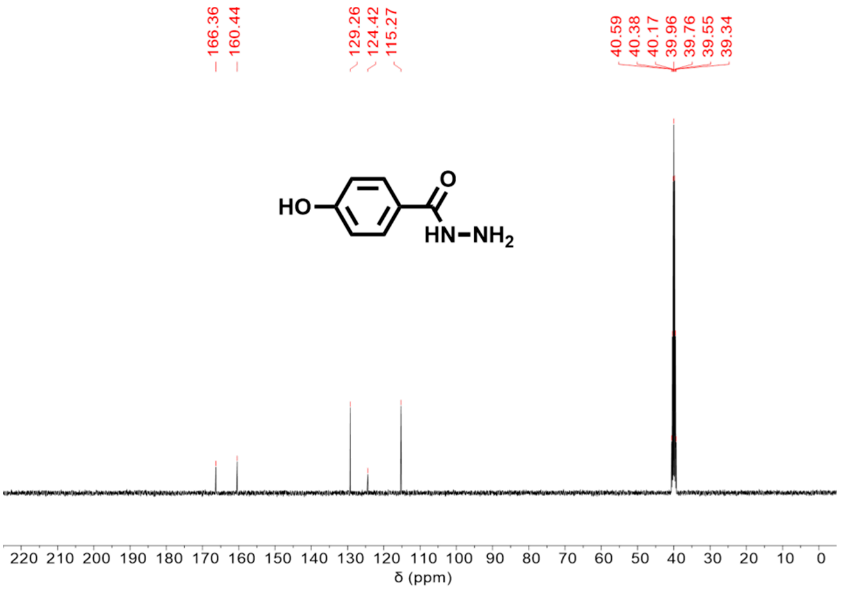

Figure S2. ^{13}C NMR spectrum (DMSO-d_6, 100 MHz, 298 K) of **1**.

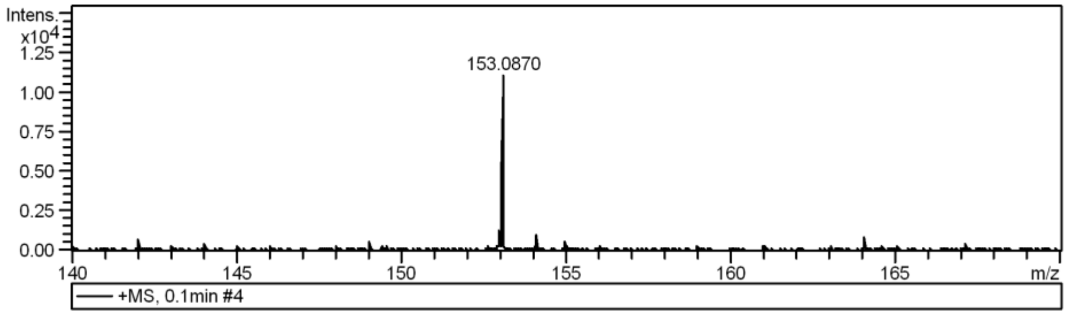

Figure S3. HR-ESI$^+$-MS spectrum of **1**.

Characterization of compound 2.

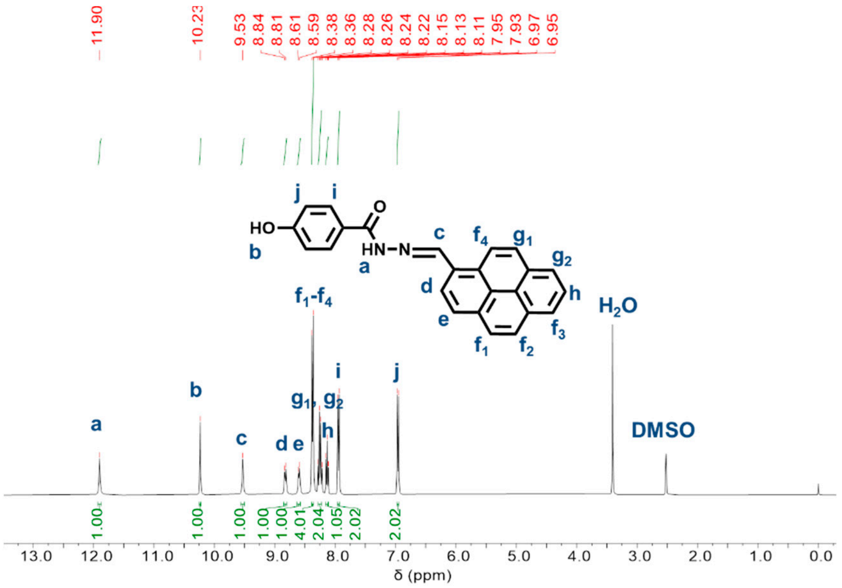

Figure S4. ^{1}H NMR spectrum (DMSO-d_6, 400 MHz, 298 K) of **2**.

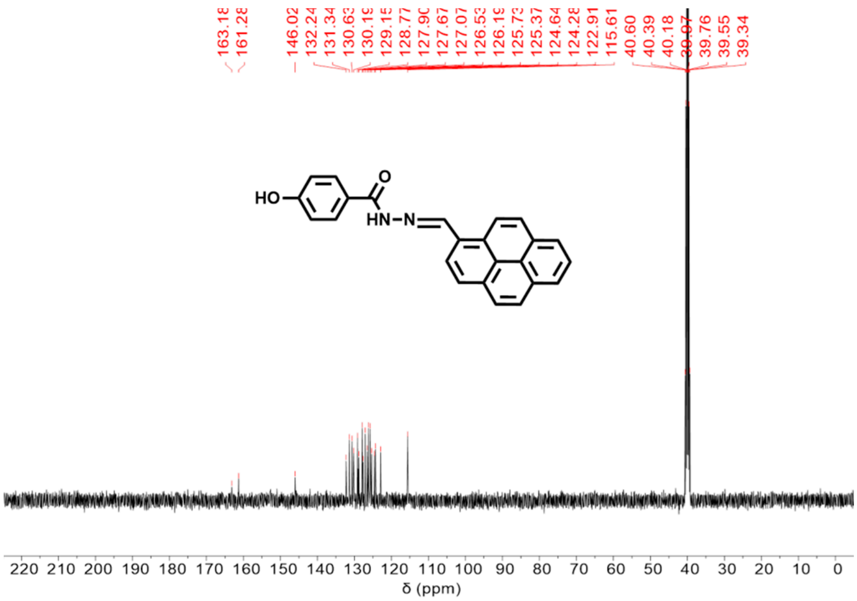

Figure S5. ^{13}C NMR spectrum (DMSO-d_6, 100 MHz, 298 K) of **2**.

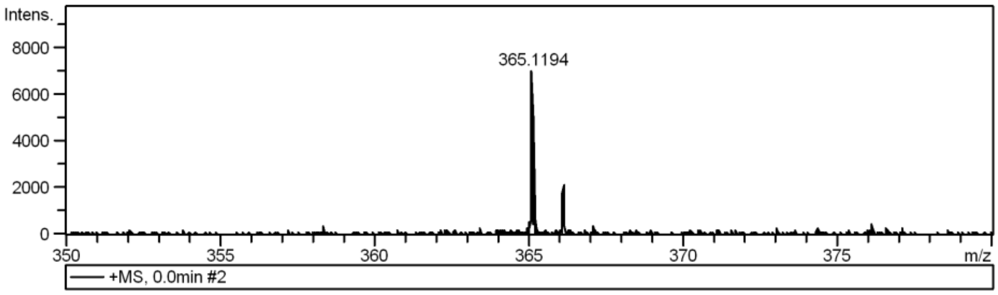

Figure S6. HR-ESI$^+$-MS spectrum of **2**.

Characterization of compound 3.

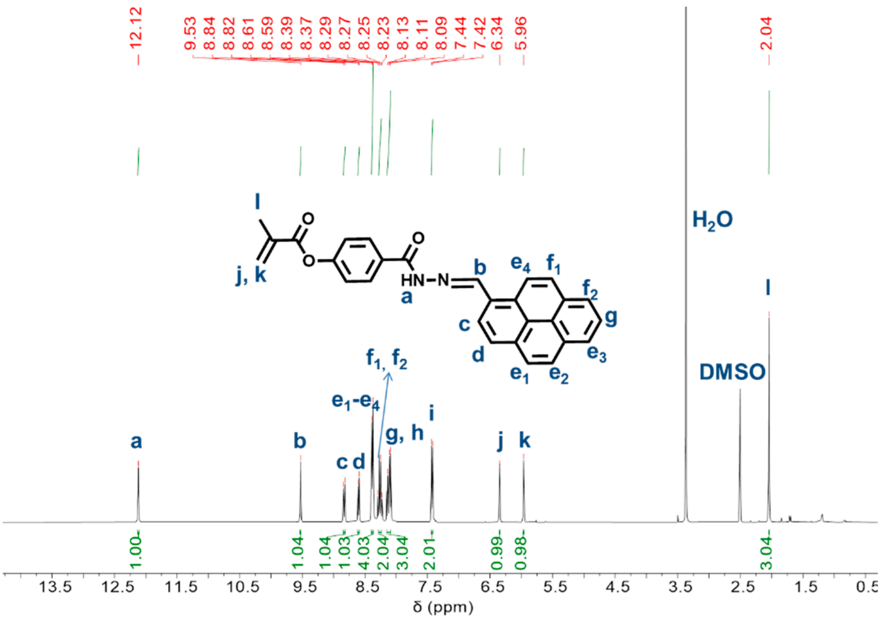

Figure S7. ^{1}H NMR spectrum (DMSO-d_6, 400 MHz, 298 K) of **3**.

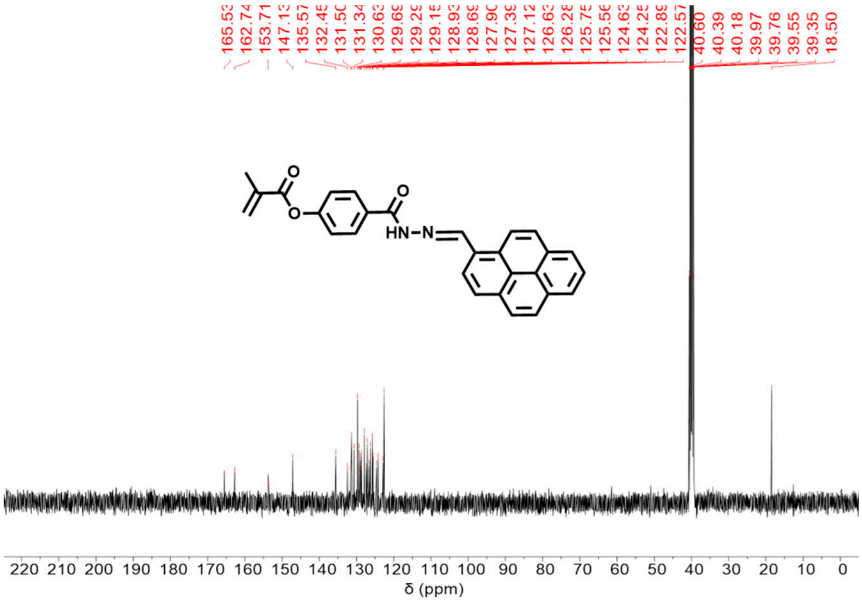

Figure S8. ^{13}C NMR spectrum (DMSO-d_6, 100 MHz, 298 K) of **3**.

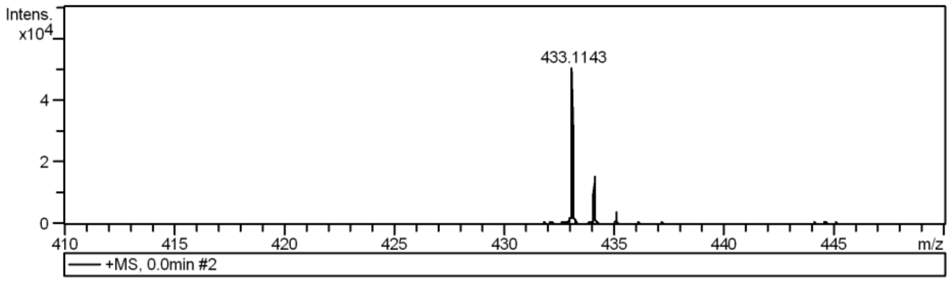

Figure S9. HR-ESI$^+$-MS spectrum of **3**.

2. Synthesis and characterization of compound 4

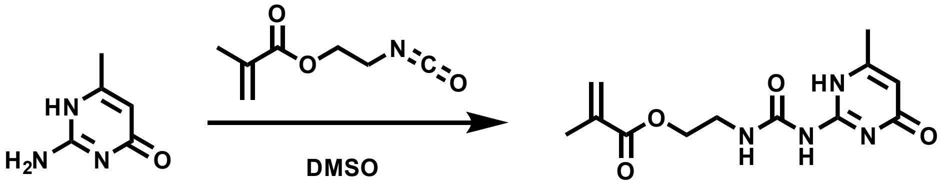

4

Scheme S2. Synthetic route of compound **4**.

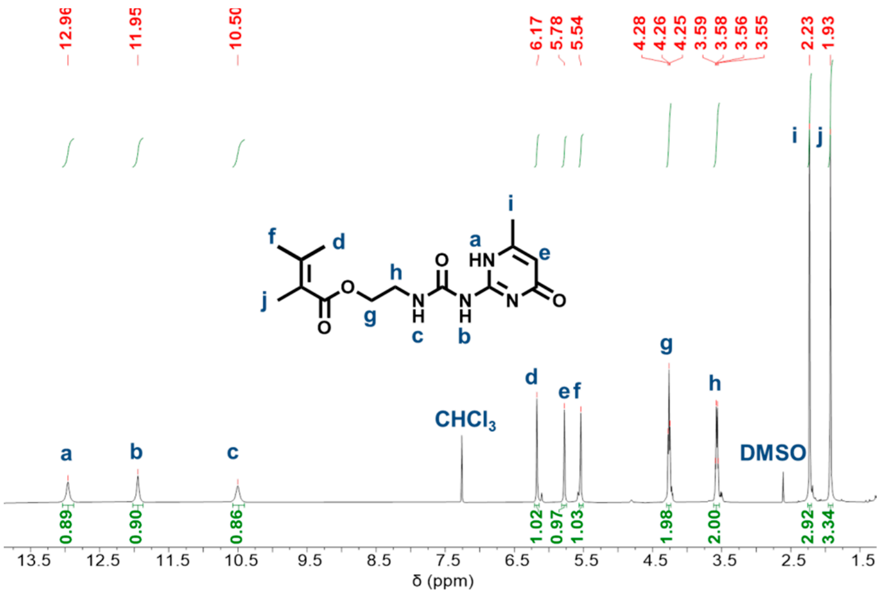

Figure S10. ^{1}H NMR spectrum ($CDCl_3$, 400 MHz, 298 K) of **4**.

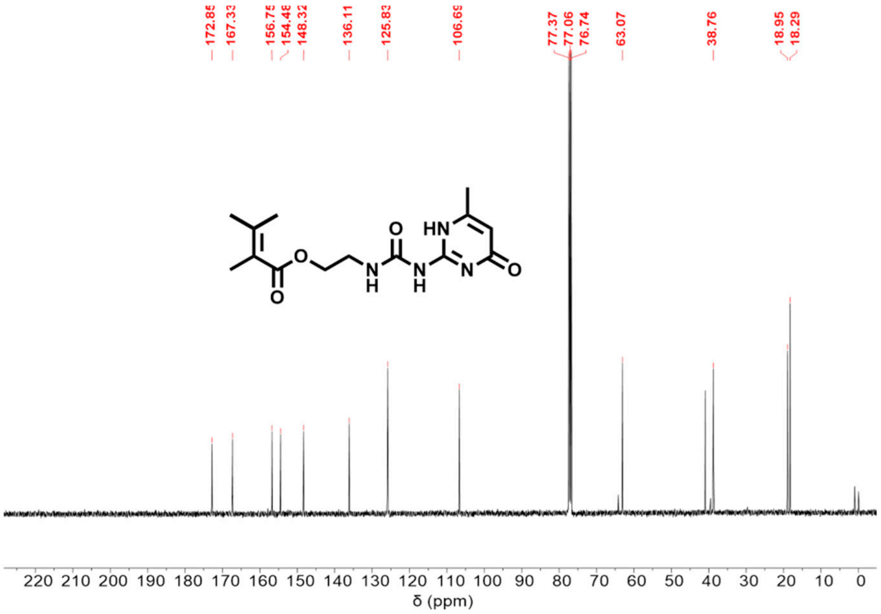

Figure S11. ^{13}C NMR spectrum ($CDCl_3$, 100 MHz, 298 K) of **4**.

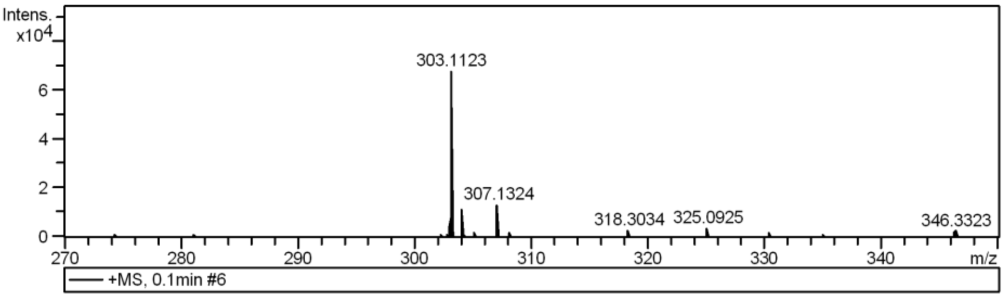

Figure S12. HR-ESI^{+}-MS spectrum of **4**.

3. Synthesis and characterization of PPMU polymer

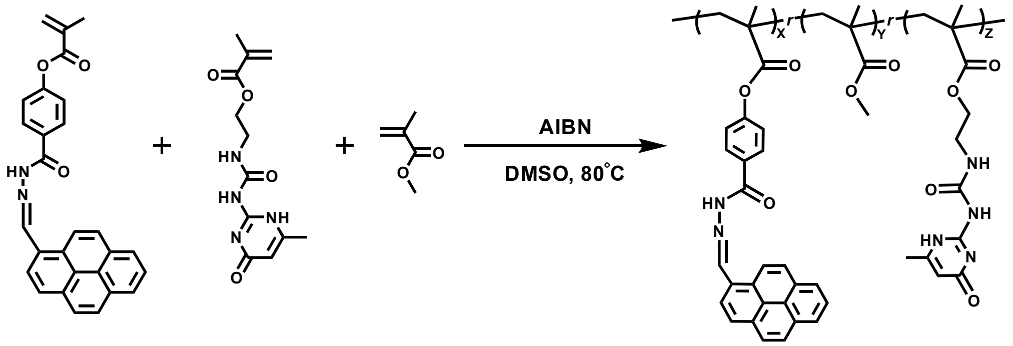

Scheme S3. Synthetic route of **PPMU** polymer.

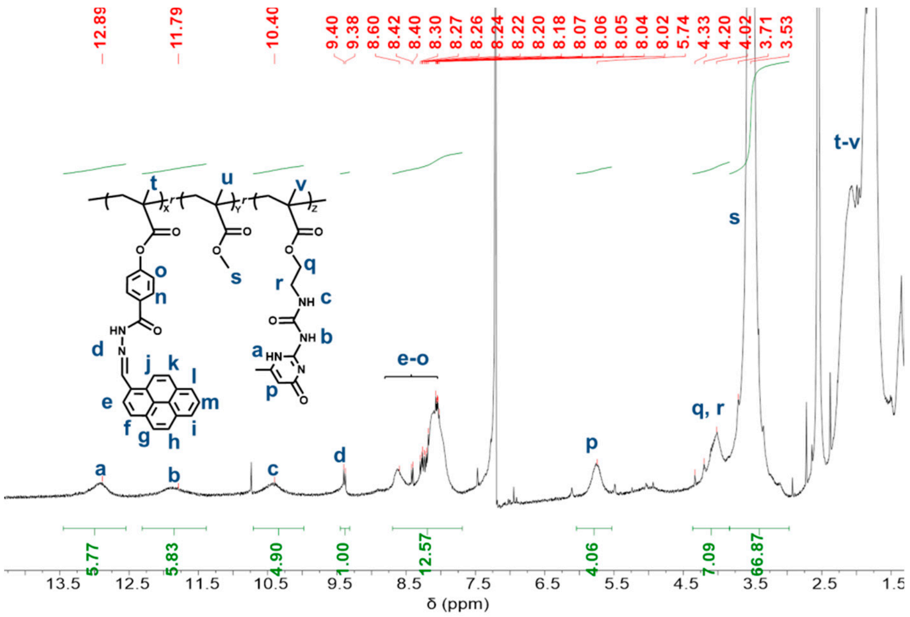

Figure S13. ^{1}H NMR spectrum ($CDCl_3$, 400 MHz, 298 K) of **PMMU** polymer.

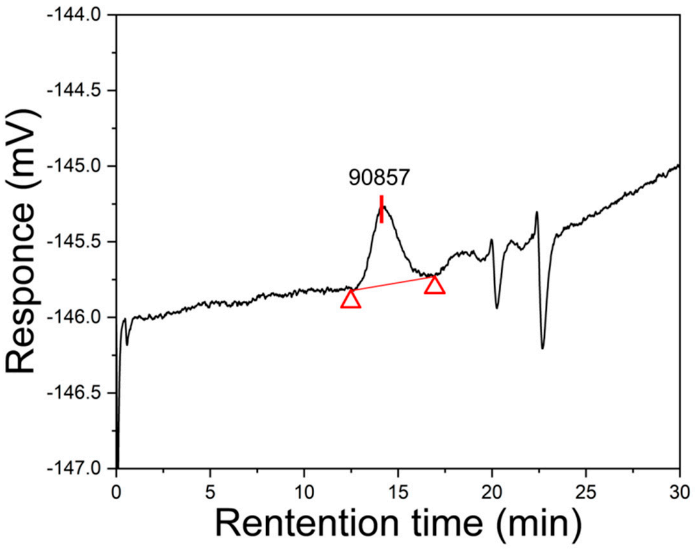

Figure S14. GPC trace of **PPMU** polymer.

M_n	M_w	M_p	M_z	$Đ$
6.79×10^4	1.19×10^5	9.09×10^4	1.99×10^5	1.75

Table S1. GPC analysis of **PPMU** polymer using conventional calculations, with polystyrene as the standard and THF as the solvent.
